# E-Cigarette Aerosol Deposition and Disposition of [^11^C]Nicotine Using Positron Emission Tomography: A Comparison of Nicotine Uptake in Lungs and Brain Using Two Different Nicotine Formulations

**DOI:** 10.3390/ph15030367

**Published:** 2022-03-17

**Authors:** Anders Wall, Sara Roslin, Beatrice Borg, Simon McDermott, Tanvir Walele, Thomas Nahde, Grant O’Connell, Joseph Thompson, Mark Lubberink, Gunnar Antoni

**Affiliations:** 1 PET Centre, Uppsala University Hospital, SE-751 85 Uppsala, Sweden; anders.wall@akademiska.se (A.W.); sara.roslin@akademiska.se (S.R.); beatrice.borg@akademiska.se (B.B.); 2 Department of Surgical Sciences, Uppsala University Hospital, Entrance 70, SE-751 85 Uppsala, Sweden; mark.lubberink@akademiska.se; 3 Department of Medicinal Chemistry, Uppsala Biomedical Centre, Uppsala University, SE-751 23 Uppsala, Sweden; 4 Imperial Brands PLC, 121 Winterstoke Road, Bristol BS3 2LL, UK; simon.mcdermott@uk.imptob.com (S.M.); taw444@hotmail.co.uk (T.W.); grant.oconnell@uk.imptob.com (G.O.); joseph.thompson@uk.imptob.com (J.T.); 5 Reemtsma Cigarettenfabriken GmbH (Part of Imperial Brands PLC), Max-Born-Straße 4, 22761 Hamburg, Germany; thomas.nahde@reemtsma.de

**Keywords:** tobacco harm reduction, e-cigarettes, nicotine, carbon-11, PET, inhalation, kinetics

## Abstract

Smoking is a cause of serious disease in smokers. Electronic cigarettes, delivering aerosolized nicotine, offer adult smokers a potentially less harmful alternative to combustible cigarettes. This explorative PET/CT study investigated the distribution and deposition of inhaled [^11^C]nicotine using the *my*blu^TM^ e-cigarette with two nicotine formulations, freebase and lactate salt. Fifteen healthy adult smokers participated in the two-part study to assess the distribution and accumulation of [^11^C]nicotine in the respiratory pathways and brain. Time-activity data for the respiratory pathways, lungs, oesophagus and brain were derived. 31–36% of both inhaled tracer formulations accumulated in the lung within 15–35 s. [^11^C]Nicotine_freebase_ exhibited higher uptake and deposition in the upper respiratory pathways. For [^11^C]nicotine_lactate_, brain deposition peaked at 4–5%, with an earlier peak and a steeper decline. A different kinetic profile was obtained for [^11^C]nicotine_lactate_ with lower tracer uptake and accumulation in the upper respiratory pathways and an earlier peak and a steeper decline in lung and brain. Using nicotine lactate formulations in e-cigarettes may thus contribute to greater adult smoker acceptance and satisfaction compared to freebase formulations, potentially aiding a transition from combustible cigarettes and an acceleration of tobacco harm reduction initiatives.

## 1. Introduction

Smoking tobacco is a cause of serious diseases in smokers, including lung cancer, heart disease and emphysema. The best action adult smokers can take to reduce these risks is to stop smoking or using nicotine products completely. However, for adult smokers who are neither interested nor willing to quit smoking, a growing number of regulators and public health bodies posit that encouraging them to transition to using non-combustible nicotine products, that are substantially less harmful than inhaled tobacco smoke, is the next best option [1,2]. This is the basis of tobacco harm reduction, one of the most promising global public health policies available with the potential to save many lives if fully embraced. Electronic cigarettes (e-cigarettes) are one such category of potentially reduced harm alternatives for adult smokers that would otherwise continue to smoke.

E-cigarettes are battery-powered devices that deliver aerosolised nicotine (if included in the formulation), propylene glycol and/or glycerol, and may contain flavorings from an e-liquid of known chemical composition. They do not contain tobacco nor require combustion and are available in two principal configurations: “open” systems, which can be refilled by the consumer (e.g., tank systems) or “closed” systems (e.g., replaceable cartridges or disposables pre-filled by manufacturers). When the user takes a puff on the product or activates via a push button, a heating element is activated, converting the e-liquid into an aerosol that the user inhales. A recent Cochrane Review concluded that (1) e-cigarettes help adult smokers to stop smoking (even among those who do not intend to quit smoking) and are more effective than traditional nicotine replacement therapies (NRT) or willpower alone, and (2) and are not associated with serious unwanted effects or harm with up to 2 years of usage [3]. As e-cigarettes do not contain tobacco, nor do they involve any combustion, many of the toxicants found in combustible tobacco smoke are either absent or present at substantially reduced levels in e-cigarette aerosols [3,4,5]. Moreover, clinical biomarkers of exposure (BoE) studies with adult smokers have shown that, compared to continued combustible tobacco smoking, adult smokers who transition to e-cigarettes experience rapid and substantial reductions in exposure to cigarette smoke toxicants [3,6,7,8,9,10].

Adult smokers are more likely to transition to less harmful products such as e-cigarettes if the product provides a satisfying experience. Thus, to maximize the public health benefits of e-cigarettes, it is important that e-cigarettes provide adult smokers with a similar experience to combustible tobacco smoking, but in a less harmful way [11]. Traditional nicotine replacement therapy (NRT) products, such as nicotine gums, lozenges, patches and inhalators, deliver tobacco-derived nicotine much more slowly and at lower doses than combustible tobacco cigarettes [1,12,13]. In addition, the absence of the behavioral and sensorial aspects of the smoking experience using such products may explain the limited success of NRT for long-term smoking cessation [13]. It has been proposed that adapting the speed of nicotine delivery from e-cigarettes may be key to assisting more adult smokers in fully transitioning away from combustible tobacco smoking [14]. The need for more effective e-cigarette products to provide satisfying alternatives to smoking for more adult smokers has led to the recent commercialization of e-liquids containing ‘nicotine salts’ [12,15,16].

Freebase nicotine is more volatile than nicotine salt [16]. As a result, when an e-cigarette aerosol is inhaled, the nicotine is more likely to off-gas and deposit in the mouth and upper respiratory tract, where it is then absorbed into the blood. Absorption in these regions is slower compared to combustible tobacco smoke inhalation, with pharmacokinetic studies indicating an absorption profile that more closely resembles NRT than a combustible cigarette [17]. By contrast, charged nicotine is thought to remain to a greater extent in the aerosol droplets until it reaches the alveoli of the lower respiratory tract for pulmonary absorption, akin to combustible cigarettes [18,19]. Consistent with this hypothesis, O’Connell et al. [16] investigated the pharmacokinetic profile of a closed system e-cigarette containing either freebase nicotine or different concentrations of nicotine salt (nicotine lactate) and compared these profiles to that of a combustible cigarette in a randomized, open-label, cross-over clinical trial. Of note were the differences in pharmacokinetic profile between the freebase nicotine formulation, containing 25 mg nicotine, and the nicotine lactate formulations, containing 40, 25 or 16 mg nicotine lactate. The mean values of the maximum plasma concentration (Cmax), the time to maximum nicotine concentration (Tmax), and area under the time-concentration curve (AUC) from time zero to 30 min were all higher for the different nicotine lactate formulations than for the freebase nicotine formulation. For the three nicotine lactate formulations, all used with the *my*blu™ e-cigarette device, there was also a trend of dose proportionality for the pharmacokinetic parameters measured. All three pharmacokinetic endpoints thus indicated a more effective absorption profile for nicotine with the nicotine lactate formulation, and corresponding adult smoker satisfaction scores, than the freebase nicotine formulation with the same e-cigarette device under a controlled puffing regime.

To complement this previously published pharmacokinetic study, and to gain a further mechanistic understanding of nicotine lactate deposition, an explorative, proof of concept, PET-study, using [^11^C]nicotine, was designed to investigate the above absorption hypothesis, i.e., whether nicotine lactate has a more effective uptake than a nicotine freebase formulation. This PET imaging approach was used to explore the [^11^C]nicotine deposition and disposition in the oral cavity, lungs, and brain to better understand the principal absorption route and kinetics of nicotine in vivo following aerosol inhalation from the *my*blu™ e-cigarette device.

## 2. Results

**Figure 1:**
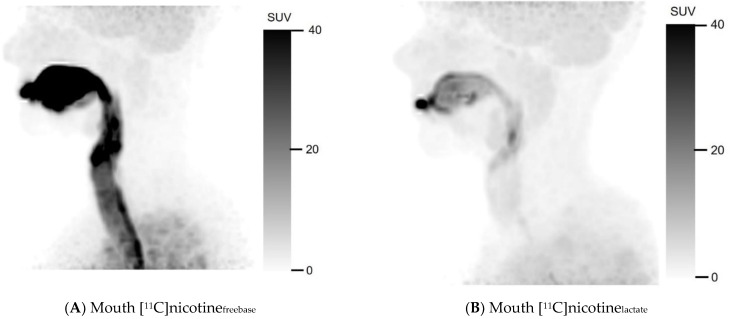

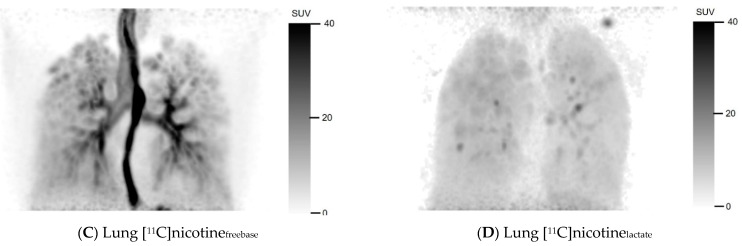
Mouth and lung summation PET images illustrating uptake of [^11^C]nicotine using freebase (**A**,**C**) and lactate (**B**,**D**) formulations, for one representative subject in each cohort. Summation was performed from first frame after inhalation without device to 10 min post inhalation.

Figure 1 shows the uptake of [^11^C]nicotine_freebase_ and [^11^C]nicotine_lactate_ in summation images comprising the first image frame where the e-cigarette was removed from the field of view until 10 min after [^11^C]nicotine administration. The freebase formulation visually showed substantial accumulation in the oral cavity and upper respiratory pathways (Figure 1A). There was also a noticeable uptake in the bronchial tree. Furthermore, [^11^C]nicotine_freebase_ uptake in the lung parenchyma beyond the bronchial tree was clearly higher than in the surrounding thorax. Part of the inhaled [^11^C]nicotine_freebase_ was also detected in the oesophagus (Figure 1C).

For the nicotine lactate formulation, a deposition of [^11^C]nicotine_lactate_ can be observed in the oral cavity after inhalation (Figure 1B) but noticeably less in the trachea compared to the freebase formulation. The lower trachea, the bronchial tree, and the oesophagus were hard to discriminate in the nicotine lactate images of the lung (Figure 1D) but some hotspots were identifiable in the bronchial tree. However, the uptake in the lung parenchyma was higher and more homogenous with [^11^C]nicotine_lactate_ compared to [^11^C]nicotine_freebase_. Even in this case, the [^11^C]nicotine_lactate_ uptake in the lungs differed notably from the surrounding thorax.

### 2.1. Discrete Early and Late Time Points

**Figure 2:**
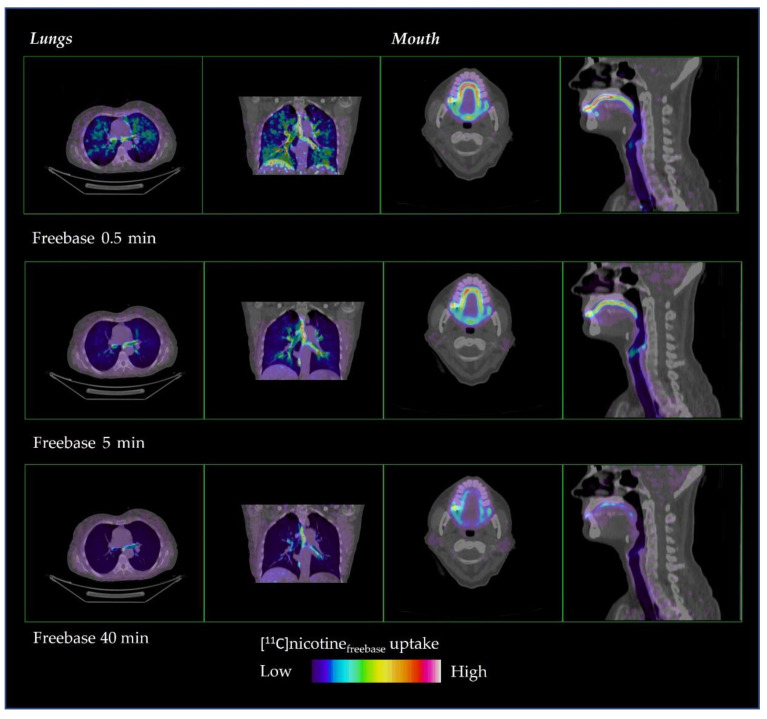
Representative distribution of [^11^C]nicotine_freebase_ in lungs and mouth at 0.5, 5 and 40 min after inhalation.

**Figure 3:**
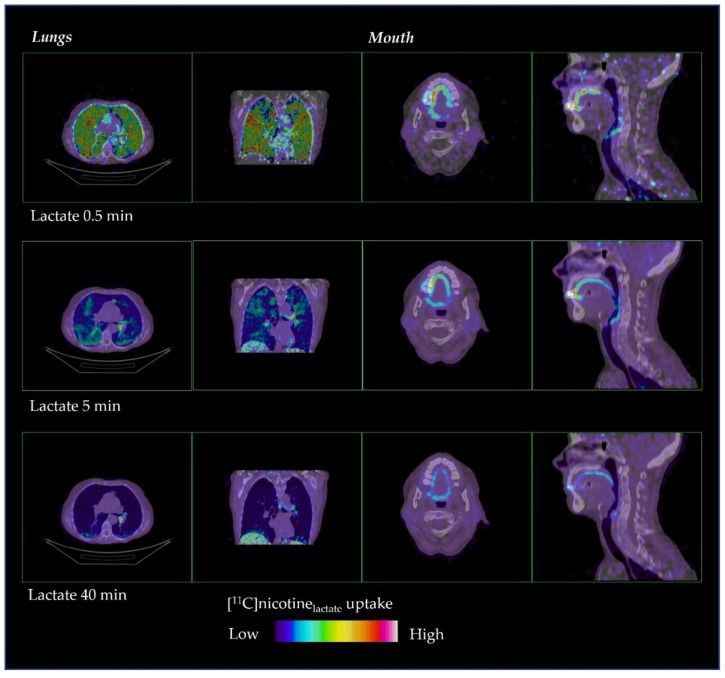
Representative distribution of [^11^C]nicotine_lactate_ in lungs and mouth at 0.5, 5 and 40 min after inhalation.

The distribution of [^11^C]nicotine over time is shown in Figure 2 and Figure 3 for freebase nicotine and nicotine lactate formulations, respectively. The same subjects are considered as in Figure 1.

[^11^C]Nicotine_freebase_ had a fast distribution through the oral cavity, upper respiratory pathways and the lung parenchyma (Figure 2). However, the freebase [^11^C]nicotine tended to remain in the oral cavity and upper respiratory pathways during the entire PET scan, up to 40 min.

A fast distribution was also observed for [^11^C]nicotine_lactate_ with a rapid passage through the oral cavity, upper respiratory pathways and the bronchial tree (Figure 3). However, the [^11^C]nicotine from the lactate formulation accumulated less in those regions compared to the freebase formulation. [^11^C]nicotine_lactate_ reached the entire lungs beyond the bronchial tree faster and to a greater extent than [^11^C]nicotine_freebase_.

### 2.2. Time Activity Data

**Figure 4:**
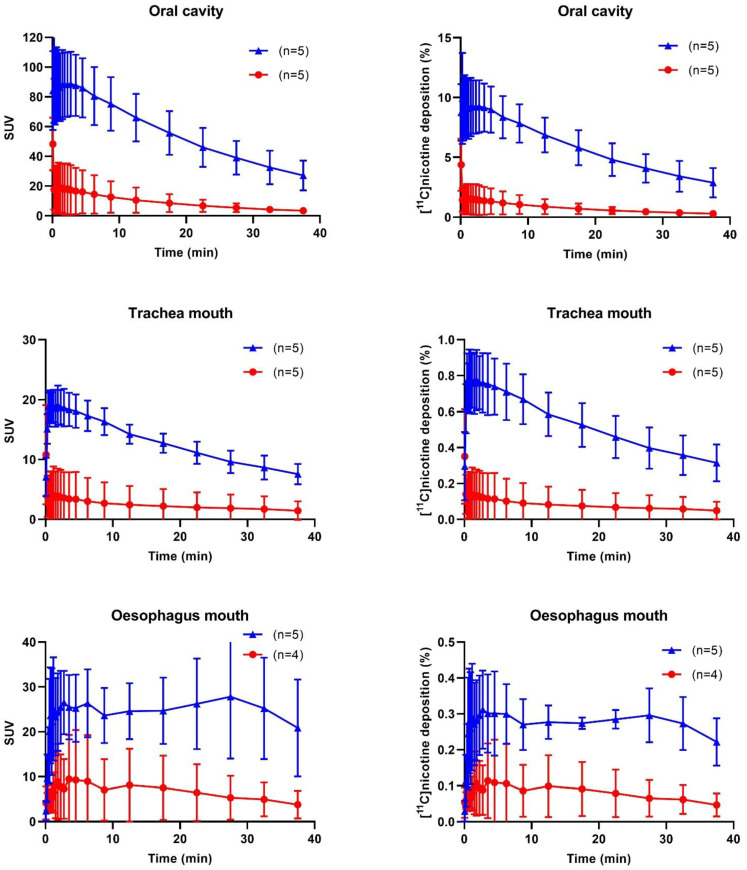

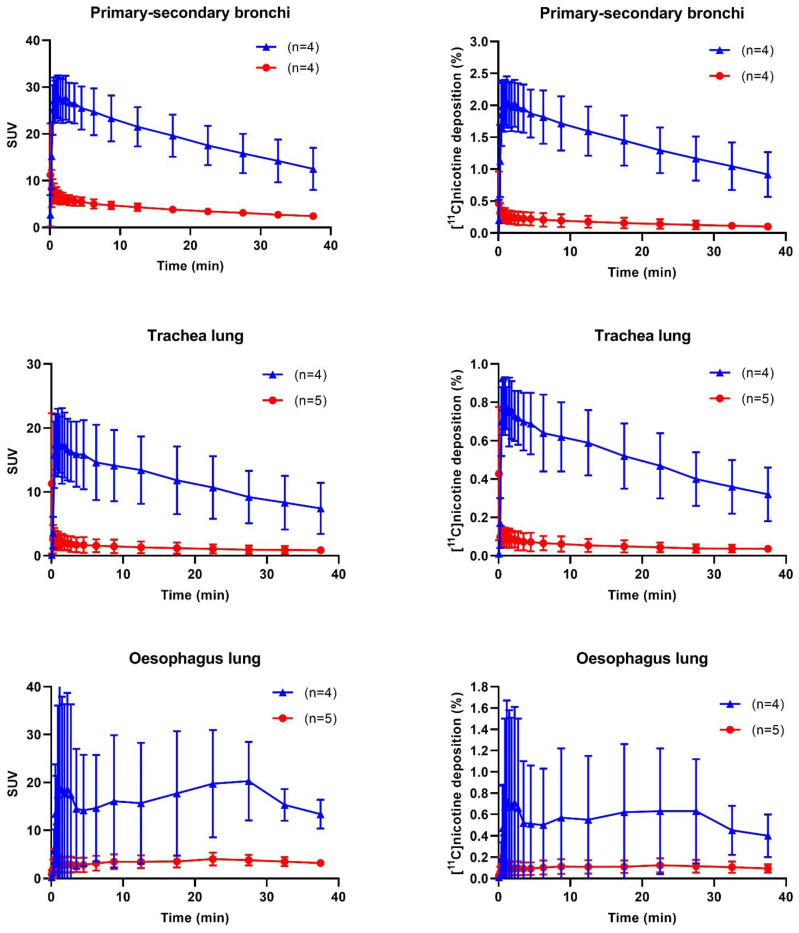

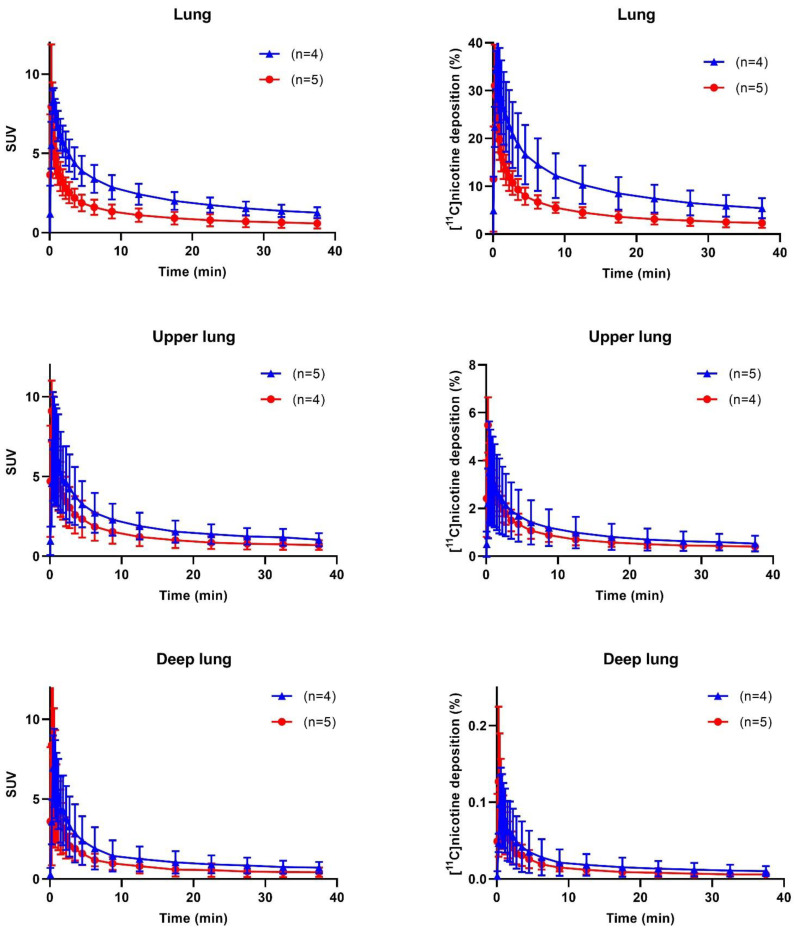
Mean ± SD of time activity for all regions, [^11^C]nicotine freebase blue plots (triangles), and [^11^C]nicotine lactate red plots (circles). Number of samples (n = 4−5) as specified in each graph.

Mean TAC data expressed as standardized uptake value (SUV) and [^11^C]nicotine deposition are presented for both freebase nicotine and nicotine lactate formulations in Figure 4. Early and local maximum values for [^11^C]nicotine were not considered as peak values. This ensures that peaks observed in the initial passage of aerosol through the VOIs are not misinterpreted as deposition. The area under the curve was also calculated from the TAC data; see Table S3 in the supplementary information. When taken together, the freebase formulation had a consistently higher deposition than the lactate formulation in most regions and for all subjects.

#### 2.2.1. Oral Cavity and Upper Respiratory Pathways

For [^11^C]nicotine_freebase_, the highest SUV was found in the oral cavity (88.7 ± 24.2, (mean ± SD), followed by the oesophagus mouth (27.8 ± 15.4)), the trachea mouth (19.1 ± 3.65), and the upper lung region (6.88 ± 3.82). The peak SUV was reached at 25 s for the upper lung and at 110 s and 165 s for the trachea mouth and for the oral cavity, respectively. By contrast, the TACs for the oesophagus mouth peaked at 1650 s (27.5 min.),

The peak SUVs for [^11^C]nicotine_lactate_ were substantially lower than [^11^C]nicotine_freebase_ for the oral cavity (18.6 ± 18.6, 79% *), oesophagus mouth (9.46 ± 11.3, 66%) and trachea mouth (3.99 ± 5.41 79% *). An exception was the upper lung region that had a slightly higher SUV (9.11 ± 2.20, 32%) for [^11^C]nicotine_lactate_. The TACs demonstrated a peak SUV at 15 s for the upper lung, 70 s for the trachea mouth, 90 s for the oral cavity, and 210 s for the oesophagus mouth. (* *p* ≤ 0.05, Mann–Whitney U-test, two-tailed, SUV freebase vs. lactate, supplementary Table S2a.)

The peak deposition of [^11^C]nicotine_freebase_ was 9.26 ± 2.44% in the oral cavity (at 165 s), 3.52 ± 2.38% in the upper lung (at 25 s), 0.78 ± 0.19% trachea mouth (at 110 s), and 0.31 ± 0.12% oesophagus mouth (at 165 s). For the [^11^C]nicotine_lactate_ the peak depositions were 5.49 ± 1.33%, in the upper lung (at 15 s), 1.52 ± 1.39% in the oral cavity (at 90 s), 0.14 ± 0.17% in the trachea mouth (at 70 s) and 0.11 ± 0.12 in the oesophagus mouth (at 210 s). The peak depositions for [^11^C]nicotine_lactate_ were lower for the oral cavity 84% **, mouth trachea 82% ** and mouth oesophagus 66% but higher for the upper lung region 56%. (** *p* ≤ 0.005 accumulation% freebase vs. lactate, Supplementary Table S2a.) The area under the curve (AUC) calculations are presented in Supplementary Table S3a.

#### 2.2.2. Trachea Lung, Oesophagus Lung, and Lung Regions

The maximum mean SUV for the freebase formulation was highest for the primary-secondary bronchi (27.9 ± 5.34), followed by the oesophagus lung (20.3 ± 9.47) and the trachea lung (17.8 ± 4.99) and the average SUV for lung and deep lung peaked at 8.38 ± 0.86 and 6.95 ± 2.34, respectively. Most regions reached their maximum SUV between 25 and 70 s after inhalation. However, the average TAC for the oesophagus lung reached a peak at 27.5 min.

For [^11^C]nicotine_lactate_, the average peak SUVs were similar in the primary-secondary bronchi, (8.29 ± 4.57 70%, lower* than freebase SUV), deep lung (8.38 ± 8.42), and lung (7.95 ± 4.39), i.e., all about 8), and lower for the oesophagus lung (4.06 ± 1.51 80% lower* than freebase SUV) and the trachea lung (2.58 ± 2.51, 86% lower * than freebase SUV). (* *p* ≤ 0.05 SUV freebase vs. lactate, Supplementary Table S2b). Similar to [^11^C]nicotine_freebase_, the average TAC for the oesophagus lung peaked at a later time point 22.5 min after inhalation of [^11^C]nicotine_lactate,_ whereas the TACs of the other regions (lung, deep lung, upper lung, trachea, primary-and secondary bronchi) peaked at 15 s after inhalation.

The peak depositions of both [^11^C]nicotine freebase and lactate formulations in the lung were 35.5 ± 9.12 and 31.0 ± 9.62%, respectively (*p* = 0.41). In the other regions (deep lung, trachea, primary-secondary bronchi, and oesophagus), the [^11^C]nicotine deposition was low, 0.10–1.98% for [^11^C]nicotine_freebase_ and 0.10–0.31% for [^11^C]nicotine_lactate_. The deposition was highest at 35–70 s after inhalation for [^11^C]nicotine_freebase_ and at 15–25 s for [^11^C] nicotine_lactate_. (* *p* ≤ 0.05 accumulation% freebase vs. lactate, Supplementary Table S2b.) Data of AUC calculations are presented in Supplementary Table S3b.

#### 2.2.3. Brain Regions

Four subjects underwent a brain [^11^C]nicotine PET scan in part B, but the [^11^C]nicotine inhalation was incomplete for two participants as only a small fraction of the radioactivity that left the e-cigarette was sufficiently inhaled. Thus, only two scans covering the whole brain, one freebase nicotine and one nicotine lactate scan, are further presented. However, the data was extended with four subjects, from part A, that had a substantial part of the inferior part of the brain covered by the PET/CT scan. Furthermore, we added the inferior brain part region to the analysis of the two complete brain scans from part B.

For the [^11^C]nicotine_freebase_ scan, the whole-brain TAC peaked with an SUV of 1.77 at 12.5 min and the highest deposition of 4.50% at 12.5 min after inhalation. The [^11^C]nicotine_lactate_ TAC revealed a peak SUV of 2.27 and the highest deposition of 5.14% at 270 s after inhalation.

**Figure 5:**
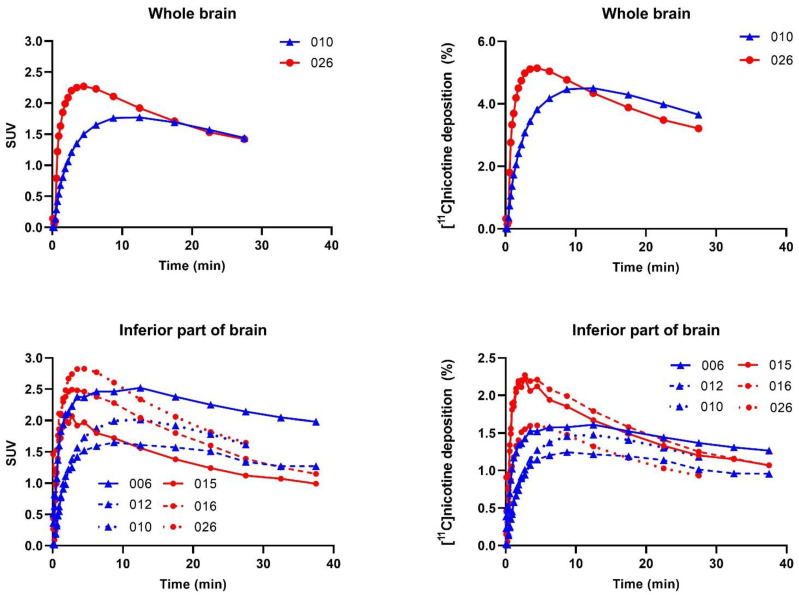
SUV versus time for all brain regions and subjects ([^11^C]nicotine_freebase_ blue plots (triangles) and [^11^C]nicotine_lactate_ red plots (circles). Subject numbers are given in each graph.

The additional analysis of the inferior part of the brain from part A confirms the findings from part B (Figure 5). For the [^11^C]nicotine_freebase_ scan, the inferior brain TAC peaked with an SUV of 2.05, and a deposition of 1.43%, both at 12.5 min after inhalation. The [^11^C]nicotine_lactate_ revealed a peak of 2.43 SUV and a peak of 2.03% deposition at 165 s after inhalation. Furthermore, the nicotine lactate formulation had a more pronounced peak in inferior brain SUV and deposition, followed by a steeper decline (Figure 5 and Supplementary Table S2c). AUC calculations are presented in Supplementary Table S3c.

### 2.3. Arterial Blood

A total of 24 [^11^C]nicotine inhalations were taken, and arterial blood was drawn from all subjects. However, it appeared that the blood sampling data was incomplete or unreliable due to problems with the arterial cannula or radioactivity measurements and coughing after inhalation. Thus, four blood datasets were excluded. The mean values and shapes of the TACs were similar for [^11^C]nicotine_freebase_ and [^11^C]nicotine_lactate_ formulations, with the highest SUV at the first time point followed by a gradual decline. The individual TACs were overlapping for both formulations, and it was not possible to discriminate any specific pattern for the formulations; see supplementary material (Figure S1) for a graph.

## 3. Discussion

Adult smokers are more likely to transition to potentially less harmful products such as e-cigarettes if the product provides a satisfying alternative to combustible cigarettes. To this end, a recent tobacco harm reduction innovation is the use of nicotine salts, such as nicotine lactate, in e-cigarette devices. Blood nicotine pharmacokinetic studies show how nicotine salts seem to better mimic the nicotine profile obtained during smoking of a combustible cigarette compared to freebase nicotine which may be important in helping more adult smokers to move away from combustible cigarette smoking [16,20,21,22,23].

This proof-of-concept study demonstrated that approximately 31–36% of the inhaled [^11^C]nicotine, freebase as well as the lactate salt formulation, was deposed in the lung within the first 15–35 s after inhalation following use of the e-cigarette, which is resemblant to [^11^C]nicotine administered via a combustible cigarette [24]. Accumulation in the other regions (deep lung, trachea, primary-secondary bronchi, and oesophagus) was low, 0.1–2.0% for the freebase formulation and 0.1–0.3% for nicotine lactate. [^11^C]Nicotine reached peak values in the brain at 4.5–12.5 min after inhalation with at most 4–5% deposition, in line with previous results [25].

For the freebase formulation, the SUV values and the accumulation were higher in the oral cavity, respiratory pathways and the oesophagus. This deposition pattern is consistent with two modes of inhalation of [^11^C]nicotine reported following the use of a nicotine inhaler [26]. Reminiscent of the blood nicotine pharmacokinetic profiles reported by O’Connell et al., 2019 [16], freebase nicotine tended to deposit more in the oral cavity and respiratory pathways than for the nicotine lactate formulation. In the current study, the [^11^C]nicotine uptake in the mouth and pharynx and the respiratory pathways was fast and measurable. Moreover, [^11^C]nicotine reached the peripheral (or deep) lung through inhalation of the e-cigarette aerosol.

The nicotine lactate formulation exhibited a faster distribution to the lungs and a higher deposition than freebase. This finding is in line with the observations from O’Connell et al. 2019 [16] that showed how nicotine lactate had a cigarette-like pulmonary blood nicotine delivery profile. In the lungs, the peak SUV and highest deposition were reached at 15 s for [^11^C]nicotine_lactate_ and 35 s for [^11^C]nicotine_freebase_. Furthermore, the nicotine lactate formulation passed the respiratory pathways faster than freebase, most likely due to less surface deposition than the freebase formulation. [^11^C]Nicotine_lactate_ also had a faster clearance from the lungs, which may indicate that the lactate formulation has a quicker and more complete redistribution into the circulating blood than the freebase formulation. This is along the line with the pharmacokinetic data reported by O’Connell et al. [16], where the mean plasma concentrations of nicotine were plotted for different nicotine strength e-cigarette products. In that study, the mean plasma nicotine concentration for the nicotine salt formulations was reached faster compared to the mean plasma nicotine concentration of the nicotine freebase comparator.

The data shows that 4–5% of the inhaled [^11^C]nicotine was in the brain at 4.5 and 12.5 min after the inhalation of the lactate and freebase [^11^C]nicotine formulation, respectively, indicating slower kinetics for [^11^C]nicotine_freebase_. Mukhin et al. [27] reported a difference in brain nicotine accumulation between men and women, and whilst it cannot be ruled out that the differences observed here are due to sex rather than the formulations, the present study was not designed to investigate differences in sex. The additional analysis of the inferior part of the brain of four subjects in part A confirmed that SUV was higher in brain tissue than in the arterial blood 300 s after inhalation (see Supplemental information for blood SUV). Data shows that both formulations of [^11^C]nicotine enter and accumulate in the brain tissue via systemic blood circulation.

The [^11^C]nicotine kinetics in arterial blood was observed with discrete arterial sampling over 30 min. The blood absorption was fast for both formulations, with the highest value observable after two min, decreasing quickly to a phase with slow elimination. No differences were discernible between the two formulations, possibly due to the data collecting timepoints chosen because of the technical limitations of the blood sampling method. However, the peak, irrespective of formulation, was probably earlier than two minutes as a study comparing e-cigarettes and combustible cigarettes reported that brain [^11^C]nicotine concentrations rose quickly (23 ± 3 s) after a single puff of the e-cigarette, as did another study where [^11^C]nicotine was administered in a combustible cigarette [24,27].

The two formulations used in the present study had an identical chemical composition apart from the charged state of nicotine, rendering the pH values to be 3.98 for the lactate formulation and 9.98 for the freebase formulation (see Section 4.2). The pH of the formulated [^11^C]nicotine solutions varied between 6–8; this is most likely due to a low buffering capacity of the added reference formulations. A regional difference in distribution and deposition was still observable for the two [^11^C]nicotine formulations as the data presented in the current study showed [^11^C]nicotine in the freebase formulation deposited in the mouth and upper respiratory tract and [^11^C]nicotine in the nicotine lactate formulation deposited in the alveoli. This result is in line with the hypothesis that freebase nicotine is indeed more volatile and thus more likely to off-gas from the aerosol droplets than the less volatile nicotine salt resulting in a different nicotine deposition profile [18,19,27]. Moreover, this proof-of-concept study demonstrates that the administration of [^11^C]nicotine via e-cigarette aerosols is sufficient for the evaluation of nicotine distribution to the lungs, circulating blood, and brain.

This was a small, descriptive and explorative study conducted in a laboratory setting, that was not designed to fully evaluate freebase and nicotine salt deposition in lung and oral tissues. As such, the small number of study subjects does not allow for comprehensive statistical comparisons to be conducted. This may explain differences in individual results based on variations in e-cigarette inhalation technique and anatomy between the subjects.

## 4. Material and Methods

### 4.1. General Chemistry Information

(*S*)-Nornicotine bitartrate was bought from Pharma Synth (Tartu, EE). (*S*)-Nornicotine was obtained as freebase via basification of an aqueous solution and extraction by diethyl ether according to an established in-house method. Solutions of nicotine and nicotine lactate salt in glycerol and propylene glycol were supplied by the study sponsor. Other reagents and solvents were obtained from Sigma-Aldrich (St. Louis, MO, USA), Fresenius-Kabi, (Bad Homburg, DE, USA), Merck (Darmstadt, DE, USA) and VWR (Radnor, PA, USA) and used without further purification. The synthesis was automated using Synthia, an in-house built synthesis equipment.

### 4.2. General Formulation Information

The two nicotine formulations used, freebase nicotine and nicotine lactate, contained the same concentration of nicotine (2.5% [*w*/*w*]). Furthermore, the formulations were optimized to ensure that the freebase nicotine formulation contained a minimal amount of protonated nicotine and the nicotine lactate formulation contained a minimal amount of freebase nicotine. Both formulations were also unflavored to ensure no flavoring compounds could influence nicotine protonation and hence possibly the deposition or absorption [28]. The nicotine lactate formulation had a pH of 3.98, and the freebase formulation had a pH of 9.98.

### 4.3. Synthesis of (S)-[Methyl-^11^C]-nicotine and Filling of myblu™ E-Cigarette

(*S)*-[Methyl-^11^C]-nicotine ([^11^C]nicotine) was synthesized by methylation of 1–2 mg (*S*)-nornicotine with [^11^C]methyl iodide [29]. [^11^C]Nicotine was purified with semi-preparative HPLC, using an Agilent Infinity 1260 II system equipped with a C18 (Waters Spherisorb ODS1, 5 μm, 250 × 10 mm) column using 50 mM ammonium formate pH 3.5 and acetonitrile as the mobile phase with UV detection at 254 nm and a Bioscan flowcount radiodetector. [^11^C]Nicotine was extracted from the collected fraction by adjusting pH > 10 by the addition of a 1 M sodium carbonate solution and retained on a tC18 (plus) SPE-column, which was washed with water. The SPE-column was flushed with 30 mL of air using a syringe. [^11^C]Nicotine was then eluted with diethyl ether, dried by passage through potassium carbonate SPE-column, into a septum equipped glass vial containing 20 µL of reference formulation (either freebase or lactate salt). The solution was concentrated to about 20 µL under a stream of nitrogen and heated at 50 °C. 10 µL of sterile water and 220–300 µL of reference formulation (the exact volume depended on the radioactivity) were added to the [^11^C]nicotine-solution giving a final formulation of [^11^C]nicotine in 2.5% nicotine as freebase or nicotine lactate in glycerol and propylene glycol without flavoring. A *my*blu™ e-cigarette was specially designed with a piece of cotton wool to absorb the radioactive [^11^C]nicotine solution. Then 100 µL of the formulated [^11^C]nicotine was applied to the cotton wool, and the *my*blu™ e-cigarette was reassembled. For each experiment, two *my*blu™ devices were prepared. Determination of identity and radiochemical purity was performed by analytical reversed-phase HPLC on an Agilent 1290 Infinity II system with eluents 8.1 mM ammonium carbonate and acetonitrile and equipped with a C18 (Phenomenex Gemini NX C18 5 µm (4.6 × 100 mm)) column using UV detection at 254 nm and a Bioscan flowcount radiodetector. The radiochemical purity was >98% for all [^11^C]nicotine productions.

### 4.4. Study Design

This study was an exploratory PET-study in two parts. Part A was designed to elucidate the uptake and deposition of inhaled ^11^C-labelled nicotine in the oral cavity, respiratory pathways, lungs, and blood. Part B illuminated the uptake and deposition in the brain and blood. Two different formulations of nicotine (freebase and nicotine lactate) were aerosolized and inhaled using the *my*blu^TM^ e-cigarette. Each study subject inhaled one of the formulations. Hereafter, the *my*blu^TM^ e-cigarette is referred to as the e-cigarette. In part A, the study subjects performed two 40 min [^11^C]nicotine PET/CT scans, one over the lungs and one over the mouth and upper respiratory pathways. The time between PET/CT sessions was at least three hours to allow for decay and washout of [^11^C]nicotine. In part B, subjects had one 30 min [^11^C]nicotine PET/CT scan over the brain. [^11^C]Nicotine was administered as 1–2 puffs, inhaled as deep draws from the e-cigarette. The subjects in part B of the study underwent a 3D T1-weighted (T1w) brain volume sequence for anatomical reference on the same day as the PET/CT scan. In part A, the ultra-low dose CT (ULDCT) scan for attenuation correction initiated before the PET scan was also used for anatomical information.

### 4.5. Study Population

Fifteen healthy adult subjects (3 males and 12 females), 51–65 (58 ± 4.4 years old) with a body weight between 51 and 85 kg (average 71 kg), were enrolled in this study (see Supplementary information Table S4 for more details). The main inclusion criteria were good general health, a daily combustible cigarette smoker, aged between 50 and 65, and absence of clinically significant diseases or disorders (as judged by the Investigator’s assessment of their medical history). Blood pressure and pulse rate measurements, 12-lead electrocardiogram (ECG), and clinical laboratory tests were carried out. All subjects were free from clinically significant diseases affecting the respiratory tract or any condition that may influence the study’s results, or the subject’s ability to participate. Furthermore, they had no history of alcoholism or substance abuse. Female study subjects were of non-childbearing potential, i.e., post-menopausal, sterilised or verified by follicle-stimulating hormone and oestradiol measurements in blood. For part B, subjects with conditions contraindicating magnetic resonance imaging (MRI) were excluded.

### 4.6. Positron Emission Tomography and Magnetic Resonance Tomography

The subject was positioned with the scanner over the lungs (A, 1st session), oral cavity (A, 2nd session), or brain (B). The PET scans were performed on a digital Discovery MI PET/computed tomography (CT) system (GE Healthcare, Waukesha, WI, USA). The scanner enables the acquisition of 89 contiguous image planes (slices) with a 25 cm axial field of view, which allows imaging of the entire lung. The first two subjects had their scans on a 20 cm axial field of view scanner, prior to the upgrade to 25 cm.

First, an ultra low dose CT attenuation correction scan (ULDCT120 kV, 10–20 mA, noise index 170) was obtained, whereupon [^11^C]nicotine (MBq) was inhaled and a PET acquisition (6 × 10 s, 3 × 20 s, 2 × 30 s, 2 × 60 s, 2 × 150 s and 4−6 × 300 s), totaling 30 and 40 min for part B and A, respectively, was started simultaneously with the [^11^C]nicotine inhalation. Images were reconstructed to a 256 × 256 pixel matrix with a field of view 50 and 25 cm for lung and oral cavity or brain, respectively, using time-of-flight ordered subsets expectation maximization with 3 iterations and 16 subsets, including resolution recovery and a 4-mm Gaussian post-processing filter. The time of [^11^C]nicotine inhalation and inhaled amount of radioactivity were registered. The inhaled amount of radioactivity was corrected for activity remaining in the e-cigarette device after inhalation.

In part B, a 3D T1w brain volume sequence (gradient-echo, duration 272 s, 1 NEX, FOV 250 mm, slice thickness 1 mm, matrix 256 × 256, flip angle 12°, TI 450 ms) was acquired on a 3.0 Tesla MR-system (Signa PET/MR, GE Healthcare, Milwaukee, WI, USA).

### 4.7. [^11^C]Nicotine, Doses and Administration

[^11^C]Nicotine was formulated with either 2.5% freebase nicotine ([^11^C]nicotine_freebase_) or nicotine lactate ([^11^C]nicotine_lactate_). The e-cigarettes, filled with 100 µL of the specific formulation spiked with [^11^C]nicotine, were delivered just a few min before each PET/CT-scan session. [^11^C]Nicotine was administered as one to two puffs on the e-cigarette followed by inhalation of the aerosol into the lungs at the onset of the dynamic [^11^C]nicotine PET scan. For the freebase formulation, 20.0 ± 4.8 MBq (n = 10) and 27.2 ± 2.6 MBq (n = 3) was inhaled for the mouth and lung scan and for the brain scan, respectively. For the nicotine lactate formulations, the doses inhaled were 24.6 ± 5.3 MBq (n = 10) and 35.0 MBq (n = 1) for the mouth and lung scan and for the brain scan, respectively. Use of the e-cigarette device was practiced with e-cigarettes containing the same formulation used in the study but with unlabelled nicotine (2.5%, freebase or nicotine salt) on the screening day to familiarize participants with both the inhalation technique and the aerosol generated by the e-cigarette. Inhalation was repeated with empty dummy devices (nicotine free) in the morning before the first PET session.

### 4.8. Consumption Restrictions

Smoking or use of nicotine, except the ^11^C-labelled nicotine, was not allowed from midnight the day before the PET assessment until completion of the last PET assessment and departure from the clinic. Further, consumption of energy drinks was prohibited during the clinic visit, but up to five cups of coffee were allowed. Water was allowed *ad libitum*, except for 15 min before [^11^C]nicotine inhalation until the end of the PET session. Consumption of alcohol was not allowed within 48 h prior to the first PET session or during the clinic stay. A breakfast was served before the first PET session and lunch before the second PET session.

### 4.9. Radioactivity Measurement in Arterial Blood Samples

Arterial blood samples, approximately 2 mL, were collected at 2, 4, 6, 8, 10, 15, 20, and 30 min after [^11^C]nicotine inhalation. The serial discrete arterial blood samples were analysed using an in-house developed well counter detector system cross-calibrated with the PET/CT scanner. Sample weight, number of counts and measurement time were recorded and the radioactivity concentrations, corrected for physical decay to the start of [^11^C]nicotine inhalation, were calculated.

### 4.10. Generation of Volumes of Interest

Volumes of interest (VOIs) were outlined on summation images from 20 s until the end of the scan. In part A, the outlined VOIs represented: lung, deep lung, trachea, primary-secondary bronchi, oral cavity, and oesophagus. Furthermore, the inferior part of the brain was delineated for four scans, two each for freebase and nicotine lactate.

**Figure 6:**
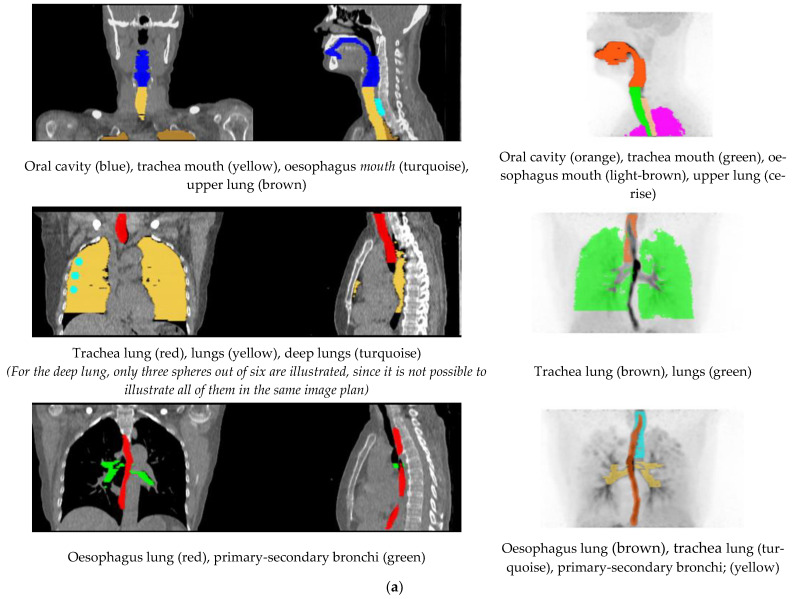

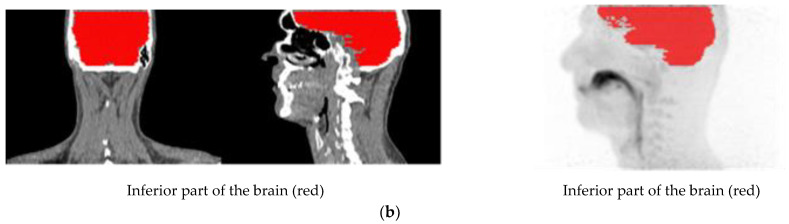
(**a**) Illustration of the delineated VOIs on mouth and lung scan. (**b**) Illustration of the delineated VOIs on inferior part of the brain.

The lung, deep lung, and trachea were delineated using the anatomical information from the ULDCT scan. Primary, secondary bronchi, oral cavity, and oesophagus were outlined on a combination of the individual’s ULDCT and [^11^C]nicotine PET summation images. The delineated VOIs were subsequently transferred to the corresponding individual dynamic [^11^C]nicotine PET scan for the generation of time-activity curves (TACs). Hermes software (GoldLx, version 2.8.0.0 and PDR, version 4.0.1), was used for VOI delineation and extraction of time-activity data. An example of the resulting VOIs is shown in Figure 6. For a detailed description of outlining of VOIs, see supplementary material.

For analysis of brain scans (part B), the PET summation image was used for a co-registration of the T1w MR volume to the summed [^11^C]nicotine PET image. The T1w MR images were segmented into grey and white matter. Both segmentation and co-registration were conducted using SPM8 (Wellcome Trust Centre for Neuroimaging Institute of Neurology, University College of London, London, UK). Subsequently, whole-brain (grey and white matter) VOIs were defined on the segmented and co-registered structural T1w images utilizing an automated probabilistic template as implemented in the PVElab software [30]. TACs were then generated with PVE-lab by projecting the whole-brain VOIs on the dynamic [^11^C]nicotine images.

TACs were converted to SUV curves by normalization to injected dose per kilogram body weight. Furthermore, the [^11^C]nicotine deposition, defined as the fraction of radioactivity measured in a delineated VOI related to the inhaled radioactivity, was calculated.

## 5. Conclusions

The exploratory study presented herein showed that the distribution of [^11^C]nicotine was fast and reached the peak value in the lungs after approximately 15 s (nicotine lactate) compared to 35 s (freebase) after [^11^C]nicotine aerosol inhalation using the e-cigarette device. Moreover, approximately 31–36% of the inhaled [^11^C]nicotine was disposed in the lung within the first 15–35 s after [^11^C]nicotine inhalation from the e-cigarette. [^11^C]nicotine_lactate_ passed the upper respiratory pathways to a greater extent than [^11^C]nicotine_freebase_, which showed a higher accumulation in the upper respiratory pathways, in line with previously reported blood nicotine delivery data.

Furthermore, the [^11^C]nicotine levels in arterial blood indicated an entry and accumulation of [^11^C]nicotine in the brain tissue via systemic blood circulation. Approximately 4–5% of the inhaled [^11^C]nicotine was distributed to the brain, where the nicotine lactate formulation showed an earlier peak and elimination compared to the freebase formulation. Although further studies are warranted as this study was based on a small number of subjects, this observation may explain greater adult smoker satisfaction and reductions in a desire to smoke combustible cigarettes with nicotine salt formulations compared to freebase in other e-cigarette studies [20,21,22,23].

## Data Availability

Data is contained within the article or supplementary materials.

## Supplementary Materials

The following supporting information can be downloaded at: https://www.mdpi.com/article/10.3390/ph15030367/s1, Detailed descriptions of VOI; Table S1: VOI volumes; Figure S1: Blood SUV; Table S2a: Peak SUV and peak accumulation mouth scans; Table S2b: Peak SUV and peak accumulation lung scans; Table S2c: Peak SUV and peak accumulation brain regions; Table S3a: AUC mouth scans; Table S3b: AUC lung scans; Table S3c: AUV brain scan regions; Table S4: Study population.

## References

[1] McNeill A., Brose L.S., Calder R., Bauld L., Robson D. (2018). Evidence Review of E-Cigarettes and Heated Tobacco Products 2018: A Report Commisioned by Public Health England. https://assets.publishing.service.gov.uk/government/uploads/system/uploads/attachment_data/file/684963/Evidence_review_of_e-cigarettes_and_heated_tobacco_products_2018.pdf.

[2] (2019). A Report by the Tobacco Advisory Group of the Royal College of Physcians. Nicotine without Smoke: Tobacco Harm Reduction. https://www.rcplondon.ac.uk/file/3563/download.

[3] Goniewicz M.L., Knysak J., Gawron M., Kosmider L., Sobczak A., Kurek J., Prokopowicz A., Jablonska-Czapla M., Rosik-Dulewska C., Havel C. (2014). Levels of selected carcinogens and toxicants in vapour from electronic cigarettes. Tob. Control.

[4] Hajek P., Etter J.-F., Benowitz N., Eissenberg T., McRobbie H. (2014). Electronic cigarettes: Review of use, content, safety, effects on smokers and potential harm and benefit. Addiction.

[5] Rudd K., Stevenson M., Wieczorek R., Pani J., Trelles-Sticken E., Dethloff O., Czekala L., Simms L., Buchanan F., O’Connell G. (2020). Chemical Composition and in Vitro Toxicity Profile of a Pod-Based E-Cigarette Aerosol Compared to Cigarette Smoke. Appl. Vitr. Toxicol..

[6] Wagener T.L., Floyd E.L., Stepanov I., Driskill L.M., Meier E., Leavens E.L., Tackett A.P., Molina N., City O., City O. (2017). Generation and Third-Generation Electronic Cigarette Users. Tob. Control..

[7] Goniewicz M.L., Smith D.M., Edwards K.C., Blount B.C., Caldwell K.L., Feng J., Wang L., Christensen C., Ambrose B., Borek N. (2018). Comparison of Nicotine and Toxicant Exposure in Users of Electronic Cigarettes and Combustible Cigarettes. JAMA Netw. Open.

[8] Shahab L., Goniewicz M.L., Blount B.C., Brown J., Mcneill A., Alwis K.U., Feng J., Wang L., West R. (2017). Nicotine, carcinogen and toxicant exposure in long-term e-cigarette and nicotine replacement therapy users: A cross-sectional study Europe PMC Funders Group. Ann. Intern. Med..

[9] O’Connell G., Graff D.W., D’Ruiz C.D. (2016). Reductions in biomarkers of exposure (BoE) to harmful or potentially harmful constituents (HPHCs) following partial or complete substitution of cigarettes with electronic cigarettes in adult smokers. Toxicol. Mech. Methods.

[10] Morris P., McDermott S., Chapman F., Verron T., Cahours X., Stevenson M., Thompson J., Chaudhary N., O’Connell G. (2021). Reductions in biomarkers of exposure to selected harmful and potentially harmful constituents following exclusive and partial switching from combustible cigarettes to myblu^TM^ electronic nicotine delivery systems (ENDS). Intern. Emerg. Med..

[11] Khoudigian S., Devji T., Lytvyn L., Campbell K., Hopkins R., O’Reilly D. (2016). The efficacy and short-term effects of electronic cigarettes as a method for smoking cessation: A systematic review and a meta-analysis. Int. J. Public Health.

[12] Caldwell B., Sumner W., Crane J. (2012). A systematic review of nicotine by inhalation: Is there a role for the inhaled route?. Nicotine Tob. Res..

[13] Farsalinos K. (2018). E-cigarettes: An aid in smoking cessation, or a new health hazard?. Ther. Adv. Respir. Dis..

[14] Schroeder M.J., Hoffman A.C. (2014). Electronic cigarettes and nicotine clinical pharmacology. Tob. Control.

[15] DeVito E.E., Krishnan-Sarin S. (2017). E-cigarettes: Impact of E-Liquid Components and Device Characteristics on Nicotine Exposure. Curr. Neuropharmacol..

[16] O’Connell G., Pritchard J.D., Prue C., Thompson J., Verron T., Graff D., Walele T. (2019). A randomised, open-label, cross-over clinical study to evaluate the pharmacokinetic profiles of cigarettes and e-cigarettes with nicotine salt formulations in US adult smokers. Intern. Emerg. Med..

[17] Fearon I.M., Eldridge A.C., Gale N., McEwan M., Stiles M.F., Round E.K. (2018). Nicotine pharmacokinetics of electronic cigarettes: A review of the literature. Regul. Toxicol. Pharmacol..

[18] Lechuga-Ballesteros D., Kuo M., Song Y., Bueche B. (2006). Aerosolizable Formulation Comprising Nicotine. US Patent.

[19] Pankow J. (2001). A Consideration of the Role of Gas and Particle Partitioning. Chem. Rearch Toxicol..

[20] Hajek P., Pittaccio K., Pesola F., Myers Smith K., Phillips-Waller A., Przulj D. (2020). Nicotine delivery and users’ reactions to Juul compared with cigarettes and other e-cigarette products. Addiction.

[21] Phillips-Waller A., Przulj D., Smith K.M., Pesola F., Hajek P. (2021). Nicotine delivery and user reactions to Juul EU (20 mg/mL) compared with Juul US (59 mg/mL), cigarettes and other e-cigarette products. Psychopharmacology.

[22] Ebajemito J.K., McEwan M., Gale N., Camacho O.M., Hardie G., Proctor C.J. (2020). A randomised controlled single-centre open-label pharmacokinetic study to examine various approaches of nicotine delivery using electronic cigarettes. Sci. Rep..

[23] Yingst J.M., Hrabovsky S., Hobkirk A., Trushin N., Richie J.P., Foulds J. (2019). Nicotine Absorption Profile Among Regular Users of a Pod-Based Electronic Nicotine Delivery System. JAMA Netw. Open.

[24] Berridge M.S., Apana S.M., Nagano K.K., Berridge C.E., Leisure G.P., Boswell M.V. (2010). Smoking produces rapid rise of [11C]nicotine in human brain. Psychopharmacology.

[25] Rose J.E., Mukhin A.G., Lokitz S.J., Turkington T.G., Herskovic J., Behm F.M., Garg S., Garg P.K. (2010). Kinetics of brain nicotine accumulation in dependent and nondependent smokers assessed with PET and cigarettes containing11C-nicotine. Proc. Natl. Acad. Sci. USA.

[26] Bergström M., Nordberg A., Lunell E., Antoni G., Långström B. (1995). Regional deposition of inhaled 11C-nicotine vapor in the human airway as visualized by positron emission tomography. Clin. Pharmacol. Ther..

[27] Solingapuram Sai K.K., Zuo Y., Rose J.E., Garg P.K., Garg S., Nazih R., Mintz A., Mukhin A.G. (2020). Rapid Brain Nicotine Uptake from Electronic Cigarettes. J. Nucl. Med..

[28] El-Hellani A., El-Hage R., Baalbaki R., Talih S., Shihadeh A., Saliba N. (2015). Quantification of free-base and protonated nicotine in electronic cigarette liquids and aerosol emissions. Chem. Res. Toxicol..

[29] Långström B., Antoni G., Halldin H., Svärd H., Bergson G. (1982). Synthesis of some 11C-labelled alkaloids. Chem. Scr..

[30] Svarer C., Madsen K., Hasselbalch S.G., Pinborg L.H., Haugbol S., Frokjaer V.G., Holm S., Paulson O.B., Knudsen G.M. (2005). MR-based automatic delineation of volumes of interest in human brain PET images using probability maps. Neuroimage.

