# Supplementary material for: E-Cigarette Aerosol Deposition and Disposition of [11C]Nicotine Using Positron Emission Tomography: A Comparison of Nicotine Uptake in Lungs and Brain Using Two Different Nicotine Formulations
Source: Pharmaceuticals (Basel). 2022 Mar 17;15(3):367. doi: 10.3390/ph15030367 (PMC8950566; doi:10.3390/ph15030367)
Supplement: Supplementary file 1 [file pharmaceuticals-15-00367-s001.zip › pharmaceuticals-1589787-supplementary.pdf]

Supplemental information

Detailed descriptions of VOI

Mouth regions

Generally, four VOIs were delineated for the mouth scans. In four cases also the lower brain was delineated. For some delineation details, see below.

Oral cavity (including pharynx)

- Delineation on the summation PET-image guided by [^{11}C]nicotine uptake and for anatomy using ULDCT
- Delineated from the top of the oral cavity down to the slice comprising the mid corniculate cartilage.
- Mean volume ± SD: 67.5 ± 17.5

Trachea mouth

- Delineation on the ULDCT image-guided anatomy and adjusted for [^{11}C]nicotine uptake using the PET summation image.
- Trachea was delineated from the level of vocal cords and downwards.
- The size of VOIs was enlarged to cover the uptake in the tracheal walls, and adjusted to match the PET-uptake
- Mean volume ± SD: 26.1 ± 4.7

Oesophagus mouth

- Delineation on the PET summation image guided by [^{11}C]nicotine uptake and adjusted for anatomy using ULDCT
- Delineated in all the image planes covering esophagus using fixed-size circles (1.2 cm in diameter)
- The fixed circle was not appropriate for delineation in all image slices; freehand VOIs were used in those slices.
- Mean volume ± SD: 8.4 ± 1.2

Upper lung

- Delineation on the ULDCT image, guided by anatomy
- Delineated from top of the lung and downwards, as far as lung was present in the field of view. Mean volume ± SD: 365.9 ± 128.5

Lower brain

- Delineation on the ULDCT image, guided by anatomy

- The lower brain VOI was outlined in each consecutive slice where the brain was present.
- The lower brain was delineated for two freebase and lactate scans
- Mean volume ± SD: 641 ± 159.7

Lung regions

Five VOIs were delineated for the lung scans. For some delineation details, see below.

Lung

- Delineation on the ULDCT image, guided by anatomy
- Outlined from top of the lung and downwards
- The most inferior level of the lungs, close to the diaphragm, was not outlined as substantial movements occurred due to breathing.
- Both lungs were merged to one VOI
- Mean volume ± SD: 3050 ± 624.9

Deep lung

- Delineation on the ULDCT image, guided by anatomy
- Delineated from top of the right lung and downwards. Fixed-size spheres (1.5 cm in diameter) were delineated in peripheral lung tissue, i.e., tissue free from significant blood vessels and bronchi as far as possible.
- The spheres were placed at every 10th slice (center-to-center). The first sphere was placed on a slice 5 slices below top of the lung. The last sphere was placed on a level without spillover from liver uptake.
- Mean volume ± SD: 10.6 ± 1.1

Trachea lung

- Delineation on the ULDCT image, guided by anatomy, and adjusted for [^{11}C]nicotine uptake
- Delineated from top of CT image and downwards to a slice 7-8 slices above the bifurcation of trachea.
- The size of VOIs was enlarged to cover the uptake in the tracheal walls, and adjusted to match the PET-uptake
- Mean volume ± SD: 32.6 ± 9.0

Primary-secondary bronchi

- Delineation on the PET summation image-guided by [^{11}C]nicotine uptake and adjusted for anatomy
- Outlined from 7-8 slices above the trachea's bifurcation and downwards beyond the second but not beyond the third bifurcation.
- The primary and secondary bronchi were delineated in each slice as far as PET uptake was noticed.
- Mean volume ± SD: 52.5 ± 16.7

Oesophagus lung

- Delineation on the PET summation image-guided by [^{11}C]nicotine uptake and adjusted for anatomy
- Delineated in all the image planes covering esophagus Fixed-size circles (1.2 cm in diameter) were delineated; if not appropriate freehand VOIs were delineated.
- Mean volume ± SD: 24.9 ± 2.0

For some subjects, the [^{11}C]nicotine uptake was not distinguishable for all image planes in primary-secondary bronchi and oesophagus. Only the parts of the region with distinguishable uptake were delineated.

Supplemental Table S1 VOI volumes in ml

Mouth scan	Oral_cavity	Trachea *mouth*	Oesophagus *mouth*	Upper lung	Lower_brain	Formulation
S0001	no scan	no scan	no scan	no scan	no scan	Freebase
S0002	60	28	7	127	-	Freebase
S0006	56	26	10	418	474[α]	Freebase
S0009	115	23	7	167	-	Freebase
S0012	67	32	9	482	514[α]	Freebase
S0027	76	30	9	493	-	Freebase
S0013	63	30	8	502	-	Lactate
S0015	63	14	not in FOV[†]	not in FOV[†]	882[α]	Lactate
S0016	45	25	10	387	587[α]	Lactate
S0017	68	26	8	378	-	Lactate
S0019	62	26	9	339	-	Lactate
Mean±SD freebase	74.7±21.2	27.8±3.1	8.0±1.1	337.5±158.0	494.1±20.1	
Mean±SD lactate	60.3±7.7	24.3±5.4	8.9±0.9	401.4±60.7	734.2±147.6	
Mean±SD both formulations	67.5±17.5	26.1±4.7	8.4±1.2	365.9±128.5	614.1±159.7	

Lung scan	Prim_sec_bronchi	Trachea *lung*	Oesophagus *lung*	Deep lung	lung	Formulation
S0001	46	29	22	11	3053	Freebase
S0002	no scan	no scan	no scan	no scan	no scan	Freebase
S0006	56	28	25	11	2983	Freebase
S0009	80	47	25	11	3043	Freebase
S0012	64	30	6*	9	3033	Freebase
S0027	29	31	27	11	3251	Freebase
S0013	22*	30	8*	12	3563	Lactate
S0015	28*	51	28	12	4392	Lactate
S0016	39	27	23	9	1880	Lactate
S0017	No visual uptake[‡]	31	24	11	2893	Lactate
S0019	19*	23	16*	11	2414	Lactate
Mean±SD freebase	53.5±17.0	33.0±7.0	24.9±1.7	10.3±0.7	3072.5±92.4	
Mean±SD lactate[1]	39	32.1±10.0	24.9±2.3	11.0±1.3	3028.4±878.3	

Mean±SD both formulations[1]	52.5±16.7	32.6±8.6	24.9±2.0	10.6±1.1	3050.4±624.9

*Partial; The uptake of tracer was not covering the entire structure, VOI delineated where uptake was seen
†Not in within the field of view, the subject had a long neck hence, esophagus and upper lung was not covered by scan.
α delineated in S0006, S0012 S0015 and S0016 only.
‡ No visual uptake was observed in the primary and secondary bronchus, region not possible to delineate.
1 Partial VOIs not included in mean±SD

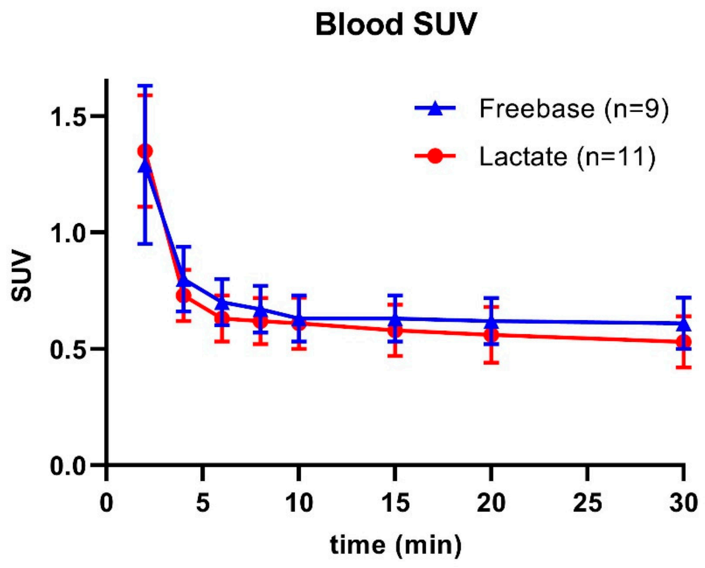

Supplemental Figure S1: Mean±SD of time activity measured in blood, [^{11}C]nicotine freebase blue plots (triangles), and [^{11}C]nicotine lactate red plots (circles). Number of samples (n=8-9) as specified in graph.

Supplemental Table S2a: peak SUV and peak accumulation% (mean± SD) mouth scans

VOI peak SUV	freebase (peak time sec)	lactate (peak time sec)	p value	n (freebase)	n (lactate)
Oral cavity	88.7 ± 24.2 (165)	18.6 ± 18.6* (90)	0.0159	5	5
Mouth trachea	19.1 ± 3.65 (110)	3.99 ± 5.41* (70)	0.0159	5	5
Mouth oesophagus	27.8 ± 15.4 (1650)	9.46 ± 11.3 (210)	0.1111	5	4
Mouth upper lung	6.88 ± 3.82 (25)	9.11 ± 2.20 (15)	0.5556	5	4
VOI peak accumulation %					
Oral cavity	9.26 ± 2.44 (165)	1.52 ± 1.39** (90)	0.0079	5	5
Mouth trachea	0.78± 0.19 (110)	0.14 ± 0.17**(70)	0.0079	5	5
Mouth oesophagus	0.31 ± 0.12 (165)	0.11 ± 0.12(210)	0.0635	5	4
Mouth upper lung	3.52 ± 2.38 (25)	5.49 ± 1.33(15)	0.2857	5	4

Freebase vs lactate * $p \le 0.05$ ** $p \le 0.01$, Mann Whitney test (two-tailed)

Supplemental Table S2b: peak SUV and peak accumulation% (mean± SD) lung scans

VOI peak SUV	freebase (time sec)	lactate (sec)	p value	n (freebase)	n (lactate)
Prim sec bronchi	27.9 ± 5.34 (70)	8.29 ± 4.57* (15)	0.0286	4	4
Lung trachea	17.8 ± 4.99 (45)	2.58 ± 2.51* (15)	0.0159	4	5
Lung oesophagus	20.3 ± 9.47 (1650)	4.06 ± 1.51* (1350)	0.0159	4	5
Lung deep lung	6.95 ± 2.34 (25)	8.38 ± 8.42 (15)	0.7548	4	5
Lung lung	8.38 ± 0.859 (35)	7.95 ± 4.39 (15)	0.5556	4	5
VOI peak accumulation %					
Prim sec bronchi†	1.98 ± 0.45 (45)	0.31 ± 0.18 (15) †	†	4	4
Lung trachea	0.79 ± 0.15 (45)	0.10 ± 0.06* (15)	0.0159	4	5
Lung oesophagus†	0.73 ± 1.09 (70)	0.12 ± 0.10† (25)	†	4	5
Lung deep lung	0.10 ± 0.05 (35)	0.13 ± 0.11 (15)	0.9048	4	5
Lung lung	35.5 ± 9.12 (35)	31.0 ± 9.62 (15)	0.4127	4	5

Freebase vs lactate * $p \le 0.05$, Mann Whitney test (two-tailed)

† The regions were partially delineated for some of the subjects, see and supplementary table 1, and detailed description of lung region VOIs in supplemental information Mann-Whitney test not done comparison and not relevant between fully delineated and partly delineated regions.

Supplemental Table S2c: peak SUV and peak accumulation% (mean± SD) brain regions

VOI peak SUV	freebase (time sec)	lactate (time sec)	n (freebase)	n (lactate)
Whole brain	1.77 (750)	2.27 (270)	1	1
Inferior part of brain	2.05 ± 0.46 (750)	2.43 ± 0.34 (165)	3	3
VOI peak accumulation %				
Whole brain	4.50 (750)	5.14 (270)	1	1
Inferior part of brain	1.43 ± 0.20 (750)	2.03 ± 0.42 (165)	3	3

Supplemental Table S3a: AUC mouth scans (mean± SD)

VOI SUV (AUC x1000)	freebase	lactate		n (freebase)	n (lactate)
Oral cavity	125.6 ± 35.9	20.62 ± 17.1**	p=0.0079	5	5
Mouth trachea	28.6 ± 4.23	5.24 ± 7.33**	p=0.0079	5	5
Mouth oesophagus	56.1 ± 20.4	14.9 ± 16.3*	p=0.0159	5	4
Mouth upper lung	4.43 ± 2.21	3.12 ± 1.62	p=0.4127	5	4
VOI accumulation % (AUC x 1000)					
Mouth oral cavity	13.1 ± 3.64	1.21 ± 0.20**	p=0.0079	5	5
Mouth trachea	1.18 ± 0.29	0.18 ± 0.23**	p=0.0079	5	5
Mouth oesophagus	0.62 ± 0.13	0.18 ± 0.17*	p=0.0159	5	4
Mouth upper lung	2.28 ± 1.61	1.80 ± 0.58	p=0.7302	5	4

Freebase vs lactate * $p \leq 0.05$, ** $p \leq 0.01$ Mann Whitney test (two-tailed)

Supplemental Table S3b: AUC lung scans (mean± SD)

VOI SUV (AUC x 1000)	freebase	lactate	p value	n (freebase)	n (lactate)
Prim sec bronchi	43.4 ± 11.4	8.85 ± 1.80*	0.0286	4	4
Lung trachea	26.2 ± 12.5	2.84 ± 2.12*	0.0159	4	5
Lung oesophagus	37.7 ± 25.0	7.85 ± 3.05*	0.0159	4	5
Lung deep lung	3.16 ± 2.09	2.16 ± 1.32	0.2857	4	5
Lung lung	5.43 ± 1.52	2.73 ± 1.13*	0.0317	4	5
VOI accumulation % (AUC x 1000)					
Prim sec bronchi†	3.20 ± 0.97	0.36 ± 0.21	- †	4	4
Lung trachea	1.15 ± 0.40	0.12 ± 0.08*	0.0159	4	5
Lung oesophagus†	1.25 ± 1.32	0.24 ± 0.15	- †	4	5
Lung deep lung	0.04 ± 0.04	0.03 ± 0.02	0.5556	4	5
Lung lung	23.2 ± 9.61	11.1 ± 2.77*	0.0159	4	5

Freebase vs lactate * $p \leq 0.05$, Mann Whitney test (two-tailed)

†The regions were partially delineated for some of the subjects, see and supplementary table 1, and detailed description of lung region VOIs in supplemental information. Mann-Whitney test not done comparison not relevant between fully delineated and partly delineated regions.

Supplemental Table S3c: AUC brain scans regions (mean± SD)

VOI SUV (AUC x 1000)	freebase	lactate	n (freebase)	n (lactate)
Whole brain	2.53	2.96	1	1
Inferior part of brain	3.70 ± 1.15	3.57 ± 0.42	3	3
VOI accumulation % (AUC)				
Whole brain	6.43	6.72	1	1
Inferior part of brain	2.58 ± 0.57	3.03 ± 0.87	3	3

Supplemental Table S4: Study population

Subject	Part of study	Formulation	Sex	Age (years)	Body weight (kg)
001	A	[^{11}C]nicotine$_{\text{freebase}}$	Male	63	85
009	A	[^{11}C]nicotine$_{\text{freebase}}$	Male	61	94
002	A	[^{11}C]nicotine$_{\text{freebase}}$	Female	62	51
006	A	[^{11}C]nicotine$_{\text{freebase}}$	Female	57	74
012	A	[^{11}C]nicotine$_{\text{freebase}}$	Female	56	68
027	A	[^{11}C]nicotine$_{\text{freebase}}$	Female	59	63
010	B	[^{11}C]nicotine$_{\text{freebase}}$	Female	64	60
011	B	[^{11}C]nicotine$_{\text{freebase}}$	Female	55	75
028	B	[^{11}C]nicotine$_{\text{freebase}}$	Female	65	64
015	A	[^{11}C]nicotine$_{\text{lactate}}$	Male	55	82
013	A	[^{11}C]nicotine$_{\text{lactate}}$	Female	56	58
016	A	[^{11}C]nicotine$_{\text{lactate}}$	Female	51	63
017	A	[^{11}C]nicotine$_{\text{lactate}}$	Female	58	65
019	A	[^{11}C]nicotine$_{\text{lactate}}$	Female	51	80
026	B	[^{11}C]nicotine$_{\text{lactate}}$	Female	54	64
